# Analyzing the reach of public health campaigns based on multidimensional aspects: the case of the syphilis epidemic in Brazil

**DOI:** 10.1186/s12889-021-11588-w

**Published:** 2021-09-06

**Authors:** Rafael de Morais Pinto, Ricardo Alexsandro de Medeiros Valentim, Lyrene Fernandes da Silva, Thaísa Góis Farias de Moura Santos Lima, Vivekanandan Kumar, Carlos Alberto Pereira de Oliveira, Cristine Martins Gomes de Gusmão, Jailton Carlos de Paiva, Ion de Andrade

**Affiliations:** 1 Federal University of Rio Grande do Norte, Natal, RN Brazil; 2 Laboratory of Technological Innovation in Health (LAIS), Natal, RN Brazil; 3 Federal Institute of Rio Grande do Norte, Natal, RN Brazil; 4 Ministry of Health, Brasilia, DF Brazil; 5 Athabasca University, Athabasca, AB Canada; 6 State University of Rio de Janeiro, Rio de Janeiro, RJ Brazil; 7 Federal University of Pernambuco, Recife, PE Brazil; 8 State Secretariat of Public Health of Rio Grande do Norte, Natal, RN Brazil

## Abstract

**Background:**

Public health campaigns aim to promote awareness, increase knowledge, and encourage a target population to adopt desirable attitudes and behaviors. Assessing their reach from a multidimensional perspective through information technology can facilitate the development of more effective campaigns in public health response.

**Methods:**

We scrutinized seven data sources from different perspectives to assess a health campaign launched in Brazil named “Syphilis No!”. This campaign is part of an Agenda for strategic actions to reduce syphilis in Brazil which includes dissemination of educommunication materials to remind people of the importance of syphilis prevention, emphasizing “test, treat and cure” concept. We developed a multidimensional analysis framework and implemented an information system to process the data from a time series perspective, and assessed the effects over time, both before and after the campaign. We descriptively analyzed data related to the campaign, including e-news, search engine activity, online courses, serological tests, medication distribution and case notification rates.

**Findings:**

Regarding search engine activity, we observed the highest volume of search during the first week of campaigns in 2018 (between November 25th and December 7th). Nevertheless, analyzing this data in a trend plot revealed sustained growth until the end of 2019. From March 2018, the amount of e-news posts related to syphilis in Brazil, indexed by Google, followed an increasing slope, with a record peak in October 2019. In addition, data showed that 12 new online courses related to syphilis disease were available on the AVASUS Platform Learning Management System (LMS), to support efforts to promote lifelong learning for health professionals, teachers, and students. These courses reached more than 22,000 students between February 2019 and September 2020. Serological test data showed that the number of tests carried out in 2019 were 375·18% more than in 2015, even accounting for population growth. Finally, starting from the middle of 2018, the syphilis case notification rates followed a decreasing curve.

**Interpretation:**

From this perspective, the “Syphilis No!” Project was a positive influence, inducing policy to fight syphilis in Brazil by supporting the implementation of a testing, treatment, and cure agenda (#TesteTrateCure). Certainly, this inference was made by analyzing multidimensional aspects and because, prior to 2018, the country had largely neglected this disease, with no records of communication actions during that period.

## Background

Public health campaigns play a strategic role in society. They communicate important information to the public [1] --promoting awareness, increasing knowledge, encouraging a target population to adopt desirable attitudes and behaviors, and contributing to individual and collective health decision-making [2]. Health campaigns are often sponsored by policy makers, non-governmental organizations (NGOs), as well as international organizations such as United Nations (UN) agencies, World Health Organization (WHO), and Pan American Health Organization (PAHO). They offer preventive recommendations concerning health problems such as sexually transmitted diseases, obesity, alcohol and tobacco addiction, obesity, as well as risks posed by automobiles, guns and pharmaceuticals [3].

Public health campaigns mainly aim to influence an individual’s behavior by proposing a change in their habits (e.g., healthy eating, physical activity, encouraging smokers to quit), and encouraging the adoption of preventive behavior (e.g., awareness of vaccination, promoting a screening service to detect sexually transmissible infections). In addition, health campaigns can be developed to help professionals, practitioners, and the general public to make informed decisions, as well as to raise awareness and understanding about critical health issues.

We reviewed literature that explores the use of information technology approaches to analyze the impact of public health campaigns. The analysis focused on identifying indicators, techniques and tools used. We realized that academic papers do not share concrete campaign data that could enable a comparison on health campaigns analysis. In addition, we observed that the studies sought to assess the impact of campaigns through quantitative [4, 5] and qualitative [6, 7] analysis, using mainly social networking services (SNS) [ 4, 6, 8] and questionnaires [5, 8–10], followed by Sexually Transmitted Infection (STI) testing [6, 8], television commercials [10, 11], campaign websites, pharmaceutical products [10], sociodemographic data [12], smoke-free restaurant laws [10], and tobacco prices changes [10]. These studies used different settings to measure the health campaign impact, which includes strengths and limitations. However, few studies have analyzed its impact through multidimensional aspects in an integrated way.

The purpose of this research is to discuss the expansive reach a public health campaign can have. Accordingly, we developed a multidimensional analysis framework considering three areas: communication, education, and epidemiological surveillance.

## Methods

This article aims to present an exploratory and descriptive analysis of data, considering three dimensions of the national Agenda for Strategic Actions to Reduce Syphilis in Brazil [13]: communication, education, and epidemiological surveillance. This analysis is supported by a multidimensional analysis framework and information system, developed by using time series decomposition analysis to assess variables of interest over time.

### Study aims

The research questions in this study are as follows:

- RQ1 - How were the campaign actions grouped and distributed over time? Reasoning: Understand whether the campaign ran throughout the entire period or if there were breaks in between. Gaps in the data may make a time series study unfeasible, as it seeks to correlate the daily scope of actions. In addition, understanding how the actions were grouped can provide insight into the type of audience sought.
- RQ2 - How has the population’s interest in the topic changed over time on the Internet? Was there an increase in spontaneous news on the topic on the Internet? Reasoning: Internet search trends may be useful to assess population health-seeking behavior, and to compare observations before and after the awareness campaign. In addition, spontaneous e-news can support campaign actions by sharing information more freely.
- RQ3 - How has the offer of courses and population engagement in technical and scientific learning on the topic changed over time? Reasoning: The engagement of health professionals, teachers and students can be seen as a strategy for the improvement of professional training, from the perspective of permanent education.
- RQ4 - How have indicators related to testing, treatment, and number of syphilis cases changed over time? Reasoning: Analyzing variations in the number of syphilis tests carried out, medication distributed and case notification rates in the pre- and post-campaign period to identify changes in epidemiological surveillance data.

### “Syphilis no!” campaign

In 2016, as part of the fight against the rising incidence of syphilis in Brazil, the Ministry of Health (MoH) prepared the Agenda for Strategic Actions to Reduce Syphilis in Brazil [13]. This document prompted a parliamentary amendment initiative of approximately $ 63,500 million, to implement a rapid response project for syphilis in 100 priority municipalities, which account for approximately 65% of the disease cases in the country.

The Agenda presented six main components (actions and activities) to facilitate syphilis decrease in Brazil: i) Educommunication; ii) Qualification of Strategic Information; iii) Strengthening the partnership between MoH and other actors; iv) Enlargement of Research Committees for Vertical Transmission of HIV (Human Immunodeficiency Virus), Syphilis and Viral Hepatitis; v) Strengthening of Health Care Networks; and vi) Rapid Response to Syphilis in Health Care Networks.

At the end of 2017, to support the objectives of the Agenda, the MoH invited state and municipal managers to align with the national strategy to fight syphilis through the “Applied research for intelligent integration aimed at strengthening health care networks for rapid response to syphilis”, known as the “Syphilis No!” Project. The general objectives of the project were to prevent pregnant women from contracting syphilis and to eliminate the transmission of congenital syphilis in Brazil.

The initiative implemented a network of Research and Intervention Supporters, to support actions to combat syphilis in priority municipalities. The role of the Supporters was to enhance action in priority municipalities and create a link between the Ministry of Health and local actors (municipal managers, health professionals and the population) [14].

In addition, MoH launched an awareness campaign to fight the syphilis epidemic in the country. The *Lembre de se cuidar – Sífilis: #TesteTrateCure* (Remember to take care of yourself - Syphilis: test, treat and cure) campaign, better known as *“Sífilis Não!”* (Syphilis No!), aimed to remind people of the importance of syphilis prevention. It highlighted the growing numbers from the epidemic, reinforced the importance of early diagnosis and appropriate emerging treatment and encouraged condom use to prevent sexual transmission of the disease. Emphasizing the importance of the “Test, treat and cure” concept, the project developed various strategies and materials, to convey the information in clear simple language directed to a general population.

The “Syphilis No!” campaign conveyed the concept of universal access to *Test, Treat and Cure,* to alert the public of available rapid syphilis test or VDRL test at any Primary Health Care (PHC) unit of the National Brazilian Health System (Sistema Único de Saúde - SUS). If tested positive for the disease, the patient could be treated at the PHC with Benzathine Penicillin medicine, and upon completion of treatment and proper medical follow-up, the patient may be considered cured.

**Fig. 1:**
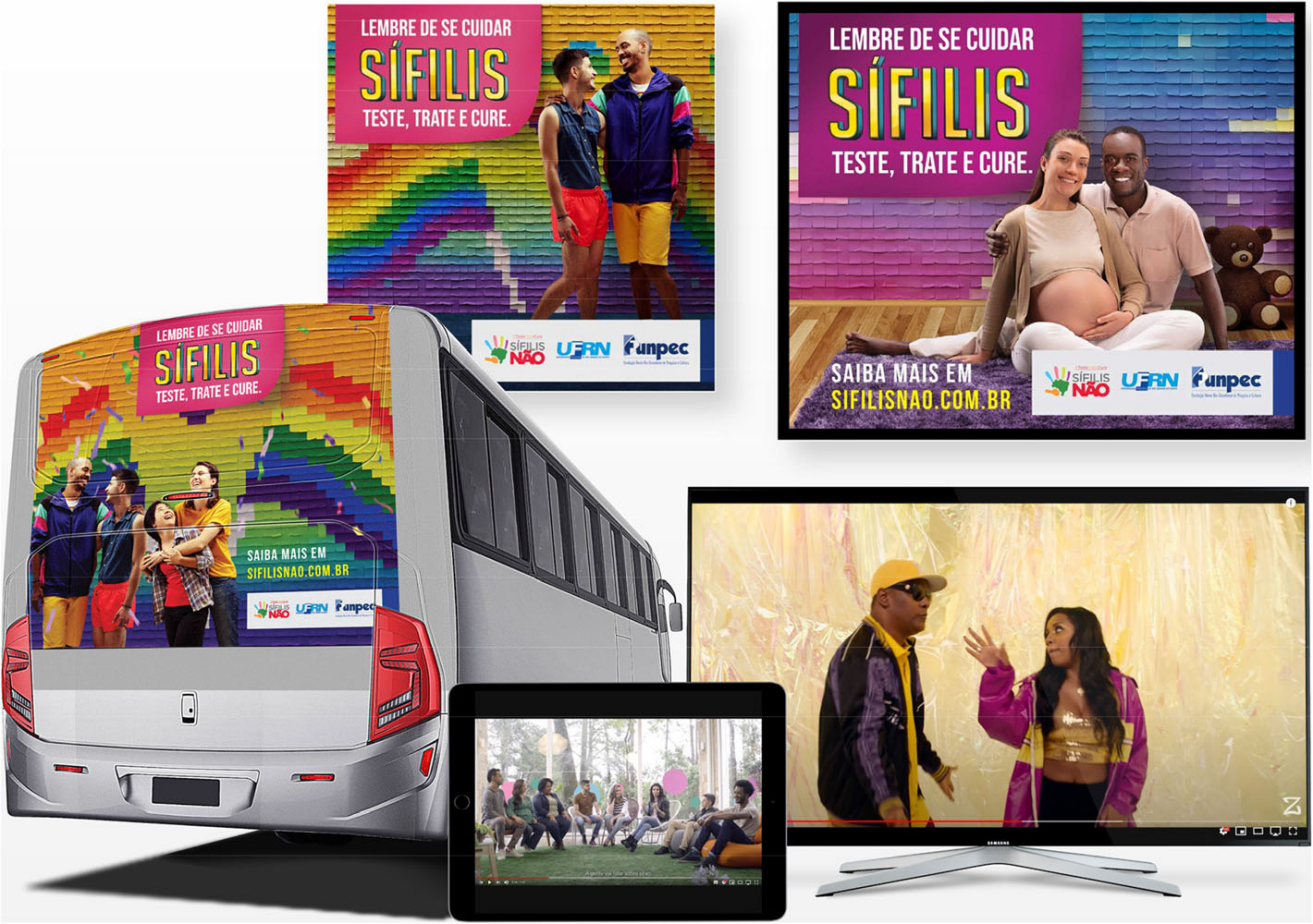
Advertisements of “Syphilis No!” Campaign produced and disseminated by television, streaming platforms, printed media, magazines, and events

This campaign ran between November 2018 to May 2019. During this period, the organizers produced and disseminated a large amount of material by television, radio, streaming platforms, printed media, magazines, live broadcasts during events, posters, informative booklets and stickers. Internet sites specifically directed toward pregnant women disseminated related content, and other content strategically emerged within news coverage, and on social networks, relationship apps, and digital pages of magazines. In addition, ten digital influencers made sponsored posts on their social networks as shown in Fig. 1.

### Study design

The exploratory study was performed through a multidimensional analysis framework. This framework is a flexible and adaptive model for the discovery and temporal analysis of the health campaign reach. It is implemented into Hermes system [15] and it manages a complete data life cycle [16] of the dimensions to be analyzed. This life cycle includes activities to: 1 - obtain data from several external APIs (Application Programming Interface) in different formats 2; − clean the data to standardize it and remove errors; 3 - transform the data into specific models, such as trends and seasonality (trends can be visualized when we see an increasing or decreasing slope in time series data, while seasonality is present when we observe a particular repeat pattern within regular intervals [17]); 4 - publish in a dashboard, so that decision makers can use it to analyze and draw relevant conclusions for planning or implementing policies in each area of interest (Hermes can serve anyone interested in analyzing campaigns’ reach); and 5 - preserve as a structured database, allowing its reuse in the future.

### Data sources usage

In a health campaign, we could analyze several data sources. Table 1 shows all data obtained by Hermes. “Syphilis No!” campaign data was obtained through an internal cooperation between Federal University of Rio Grande do Norte (UFRN) and the Ministry of Health in Brazil. Subsequently, it was manually inserted through a user interface using Hermes system. E-news data was obtained via Google Search API. Interest Over Time was collected using Google Trends. Massive Open Online Courses (MOOCs) data was collected from SUS’ Virtual Learning Environment (AVASUS) platform. Serological test data was obtained from SIA/SUS (Outpatient Information System of the Unified Health System) using web scraping. Medication distributions were obtained through SISMAT (Integrated Material Management System) provided by the MoH. Notification cases were extracted from SINAN (Information System for Notifiable Diseases), available at MoH webpage [18].

**Table 1:** Data obtained by Hermes system

Data	Source	Indicator	Acquired From	Range of Time
Campaign Data	Invoices	Daily campaigns actions	Manually Inserted into Hermes through a user interface	Nov, 2018 to Mar, 2019
E-news	Google Custom Search Engine	Number of syphilis online news posts indexed by Google	Hermes Consuming API	Jan, 2015 to Dec, 2019
Interest Over Time	Google Trends	Amount of search activity using the term ‘sífilis’ on Google	Hermes Consuming API	Jan, 2017 to Dec, 2019
Online Course Data	AVASUS	Number of Online Courses offered by the government about syphilis, and the students enrolled on them	Hermes Consuming API	Feb, 2019 to Sep, 2020
Serological tests	SIA/SUS/MoH	Number of Serological tests (non-treponemal and treponemal tests) for syphilis in PHCs	Hermes via Web Scrapy	Jan, 2017 to Dec, 2019
Medication Distribution	SISMAT/MoH	Number of Benzathine Penicillin distributed	Hermes via CVSs Files	Mar, 2016 to Dec, 2020
Case Notification	SINAN/MoH	Number of notified cases of acquired syphilis, syphilis in pregnant women, and congenital syphilis	Hermes via CVSs Files	Jan, 2015 to Dec, 2019

As each source disposes its data according to a particular pattern, there was an arduous task of cleaning the data, standardizing the scales used and filtering data about similar periods in time. Then, using time series decomposition analysis, we processed and transformed the data into trend and seasonality components, generating multiple visualizations in a single dashboard to facilitate the understanding of this information in an integrated way.

## Results

The research questions were answered according to the results produced by Hermes.

### RQ1 - how the campaign’s actions were grouped and distributed over time?

These actions were grouped through seven macro categories: Digital Media, Outdoor Media, Print Media, Radio, Social Media, Streaming Platforms, and Television. These macro categories are the same as those used in the official reports and documents of the “Syphilis No!” campaign, as follows:

- Digital Media: Messages displayed in digital media on Internet sites or apps through banners or other advertising formats, made of text, images, videos, or audio clips;
- Outdoor Media: Posters on window displays, bus stops, billboards, and other forms of media implanted in publicly accessible urban spaces;
- Print Media: Ads printed in newspapers and consumer magazines;
- Radio: Pre-recorded audio messages (spots and testimonials) delivered by radio;
- Social Media: Messages posted on social networking sites (Facebook, Instagram, and Twitter) by influencers or sponsored posts from Facebook and Instagram targeted to a specific audience (e.g. by age, educational level or occupation);
- Streaming Platforms: Sponsored videos on Youtube channels and pre-recorded audio messages delivered as ads on Spotify;
- Television: Pre-recorded videos about syphilis (spots and testimonials) delivered via television.

**Fig. 2:**
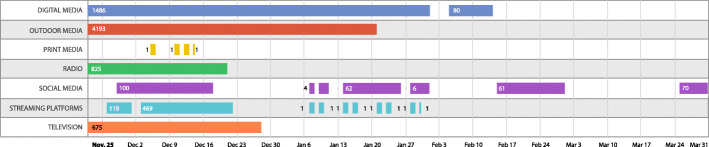
Actions’ timeline chart that depicts how the macro categories of “Syphilis No!” campaign were distributed over time

Figure 2 shows how the macro categories of “Syphilis No!” campaign were distributed over time. The actions started on November 22, 2018 and ended on March 31, 2019 with breaks i.e., during this period there were no actions every day.

Outdoor media was the macro category with the highest number of accumulated daily actions (*n* = 4193), followed by Digital Media (*n* = 1566), radio (*n* = 825), Television (*n* = 675), Stream Platforms (*n* = 596), Social media (*n* = 318), and Print Media (*n* = 4). Time gaps can be visualized in Digital Media, Print Media, Social media, and Streaming Platforms macro categories.

**Fig. 3:**
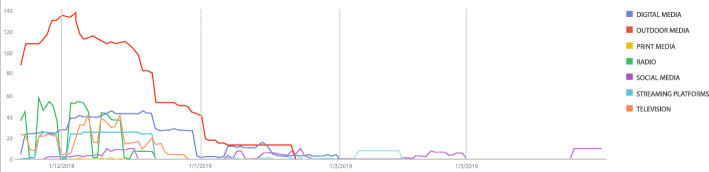
Daily number of actions per macro category

Figure 3 shows the daily number of actions per macro category. Although the actions were carried out until the end of March 2019, an abrupt reduction in the number of daily actions performed from December 23, 2018 were observed and Television, Radio, and Print Media actions ceased. Between February one and five, and between March one and 25, there were no actions.

Health campaigns are common worldwide. Nevertheless, few studies have reported actual cases and concrete campaign databases [4] such as: campaign name, ads type, topic area, target audience, period of time, country campaign location, launched/sponsor by, and amount spent. Providing the data analyzed in their studies will enable a comparison on health campaign analysis. Data related to Actions of “Syphilis No!” campaign are available under request to corresponding author, enabling researchers to explore and compare its data.

### RQ2 - how has the population’s interest in the topic changed over time on the internet? Was there an increase in spontaneous news on the subject on the internet?

To analyze the population’s interest over time, we obtain query data from the Google Trends. This data represents search activity using the term ‘sífilis’ in Brazil between January 2017 and December 2019. Google Trends provides time series data related to a specific search term carried on Google Search. It represents search interest relative to the highest point on the chart for the given region and time [19].

**Fig. 4:**
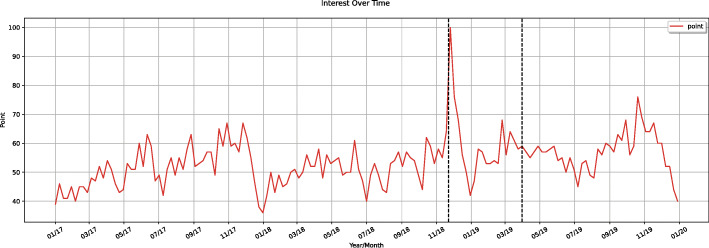
Search activity relative to the term ‘sífilis’ on Google between 2017 and 2019

Figure 4 shows the entire data week by week. The dotted vertical lines show the campaign period. The highest volume of search activity was seen during the first week immediately following actions starting November 25th and December 7th.

**Fig. 5:**
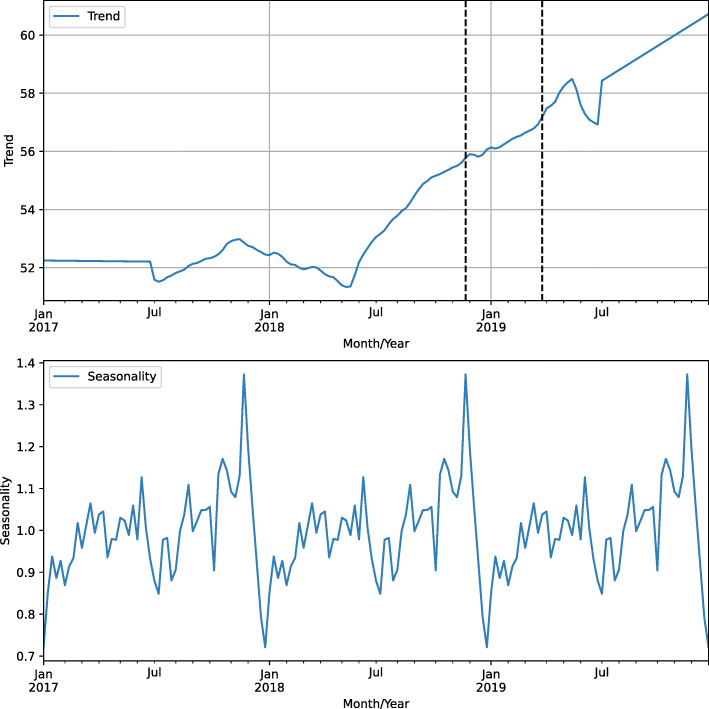
Search activity relative to the term ‘sífilis’ decomposed into trend and seasonality

Figure 5 depicts the plot of the trend and seasonality data. Trend plot shows a rising slope beginning in June 2018, as well as a smoothly decreasing slope in July 2019, which then kept rising thereafter.

In addition, it is important to mention that in May, June, and July 2018, training activities and regional seminars were held with managers from the territories and states, as well as with the Research and Intervention Supporters selected to work in 72 of the 100 priority municipalities identified within the scope of the Project, distributed in all regions of Brazil [20]. These activities were organized to promote discussions, exchange experiences and support the campaign, and certainly boosted the search for the term syphilis in Internet search engines.

In addition, seasonal plot shows a repeating pattern with peaks in searches for the term in October, November, and December, with an abrupt drop in January and February.

Regarding spontaneous news related to the topic on the Internet, the Hermes system got e-news indexed by Google, using Google Custom Search Engine (GCSE) through Google Custom Search API, between January 2015 and December 2019. GCSE is a tool that allows anyone to create and customize specific search terms for their organisation [21].

**Fig. 6:**
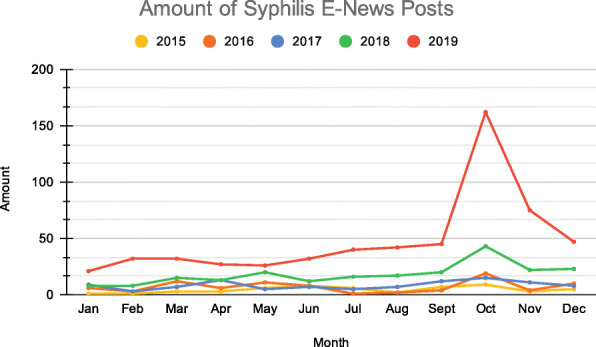
Amount of Syphilis Online News Posts indexed by Google between January 2015 and December 2019

Figure 6 shows the amount of syphilis e-news posts indexed by Google between January 2015 and December 2019, in Brazil. It shows a gradual increase in the number of e-news pieces per month and year. Every month of the year 2019 (post campaign) had more news than the months of previous years, especially the month of October, possibly because the third Saturday of October was instituted by law as the National Day to Combat Syphilis and Congenital Syphilis [22]. The law sought to encourage the participation of health professionals in the campaign activities, to emphasize the importance of proper diagnosis and treatment of syphilis.

**Fig. 7:**
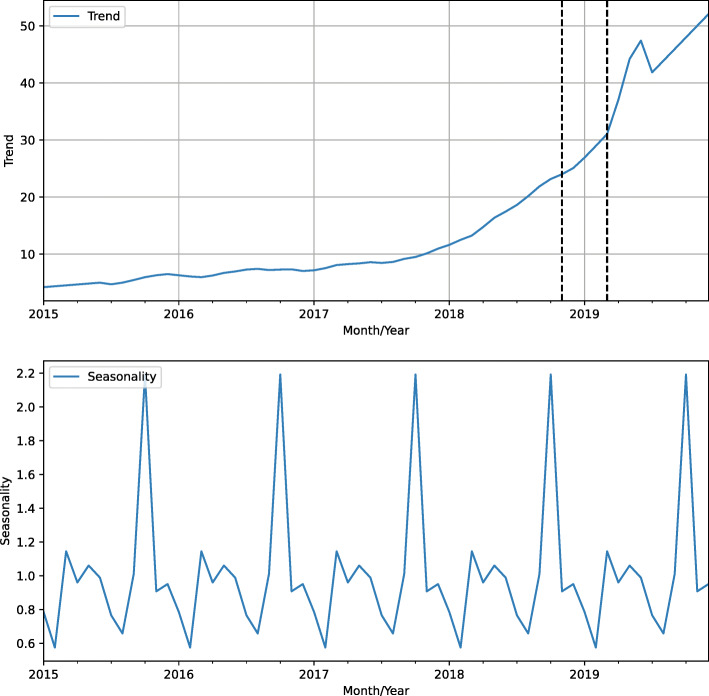
E-news indexed by Google using the term ‘sífilis’ decomposed into trend and seasonality

Decomposing these data into a trend and seasonality chart (Fig. 7), Hermes shows a rising slope from March 2018, as well as a record peak in October 2019. Regarding seasonality, there is a clear pattern of repetition with a peak in October and November, and a decline in January and February.

### RQ3 - how has the offer of courses and population engagement in technical and scientific learning on the topic changed over time?

In the perspective of lifelong learning of health professionals, the number of publications, dissertations and thesis can be seen as a powerful indicator of the interest of professors and students in the topic. However, for this study, considering the decomposition analysis of this research, other education indicators were more opportune.

Two opportune indicators related to lifelong learning are the number of MOOCs offered by the government about syphilis, and the students enrolled on them. We obtain results from the AVASUS, a platform developed by the Federal University of Rio Grande do Norte, through the Laboratory of Technological Innovation in Health (LAIS) that aims to promote distance education through online teaching and learning tools [23].

**Fig. 8:**
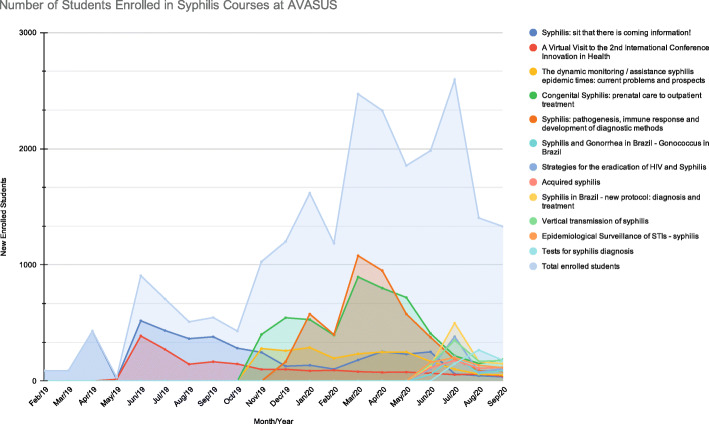
Number of Students Enrolled in Syphilis Courses at AVASUS, between February 2019 and September 2020

Figure 8 depicts the number of students enrolled in Syphilis Courses at AVASUS, between February 2019 and September 2020. In the first semester of 2019, there were only two courses on syphilis (“Syphilis: sit that there is coming information!” and “A Virtual Visit to the 2nd International Conference Innovation in Health”) with a monthly average of 310 students enrolled, considering both courses. In the second semester of 2019, three new courses were added (“The dynamic monitoring / assistance syphilis epidemic times: current problems and prospects”, “Congenital Syphilis: prenatal care to outpatient treatment”, and “Syphilis: pathogenesis, immune response and development of diagnostic methods”), raising the monthly average of students enrolled to 737. In the following semester, 2020.1, 6 new courses were added to the online platform (“Syphilis and Gonorrhea in Brazil - Gonococcus in Brazil”, “Strategies for the eradication of HIV and Syphilis”, “Acquired syphilis”, “Syphilis in Brazil - new protocol: diagnosis and treatment”, “Vertical transmission of syphilis” and “Epidemiological Surveillance of STIs - syphilis”), raising the monthly average to 1907 (which represents a growth rate of 515·16%, when compared to the same period of the previous year - 2019.1).

Finally, during the months of July, August and September 2020 (second semester), one new syphilis course was created (“Tests for syphilis diagnosis”) and the monthly average of students enrolled was 1776, which represents a growth rate of 140·98% when compared to the second half of 2019. These 12 courses have reached 22,744 students enrolled to date. Table 2 shows the enrolled students growth rate in relation to the school semesters.

**Table 2:** Enrolled students’ growth rate by school semester

School semester	Enrolled Students (monthly average)	Growth Rate
2019.1	310	..
2019.2	737	..
2020.1	1907	515·16% (compared with 2019.1)
2020.2	1776	140·98% (compared with 2019.2)

The courses with the largest number of enrolled students were “Syphilis: sit that there is coming information!” (*n* = 4284, since = Feb/19), “Congenital Syphilis: prenatal care to outpatient treatment” (*n* = 5248, since = Nov/19), and “Syphilis: pathogenesis, immune response and development of diagnostic methods” (*n* = 4487, since = Dec/19). These three courses reached 14,019 enrolled students and act exactly in the central axis of the actions of the “Syphilis No!” Project, in particular, with regards to the treatment and cure of the patients.

The engagement of health professionals, teachers and students can be considered an important indicator of transformation of health education for syphilis. As a chain reaction, health professionals with an understanding of the adverse effects of syphilis on people’s lives, guide the population to correct diagnosis, treatment, and cure of the disease. Hermes shows that educational indicators can be a valuable contribution to the strengthening of the health system.

### RQ4 - how have indicators related to testing, treatment, and number of syphilis cases changed over time?

To analyze the epidemiological surveillance dimension, we explored three indicators: serological tests, medication distribution (Benzathine Penicillin G [BPG]), and case notification rates.

Data on syphilis testing and diagnosis were collected between January 2015 and December 2019 from SIA/SUS. The choice of including two previous years to our time series approach is based on its importance for explaining the gradual increase of syphilis testing observed before 2017 in PHCs, which then becomes sustainable thereafter. We used as a core indicator, the number of Serologic tests (non-treponemal and treponemal tests) for syphilis in PHCs in Brazil, according to year.

**Fig. 9:**
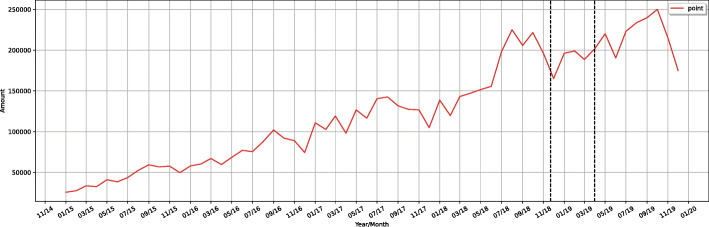
Amount of screening test performed by the primary attention service

In Fig. 9, we can observe a discrete amount of tests in 2015 (*n* = 518,859) and 2016 (*n* = 911,420) and a more obvious rise starting in 2017 (*n* = 1,448,364), 2018 (*n* = 2,068,184) that continued until late 2019 (*n* = 2,533,571). An increase in syphilis tests in Brazil is evident from 2015. These were already occurring gradually and sustainably.

To analyze the increase in testing, it is necessary to verify the population growth in the same period. Therefore, Table 3 shows the population per year, the number of tests performed, testing rate per 1000 inhabitants, the increase in the testing rate considering the population and the percentage of increase.

**Table 3:** Performed tests by year and increasing rate per 1000 inhabitants

Year	Population	Tests performed	Testing rate per 1000 inhabitants	Increased testing per 1000 inhabitants considering population size compared to 2015	Percentage increase in testing per 1000 inhabitants considering population size compared to 2015
2015	204,500,000	518,859	2·54	..	..
2016	206,200,000	911,420	4·42	1·74	74·21%
2017	207,800,000	1,448,364	6·97	2·75	174·71%
2018	209,500,000	2,068,184	9·87	3·89	289·09%
2019	210,147,000	2,533,571	12·06	4·75	375·18%

Thus, using the year 2015 as baseline, the most significant increase is observed in the years 2018 (289·09%) and 2019 (375·18%), even accounting for the country’s population growth. This means that 3·89 times more individuals were tested in 2018 and 4·75 times more in 2019, across the country. These last 2 years stand out, as they fall within the period of execution of the “Syphilis No!” Project.

**Fig. 10:**
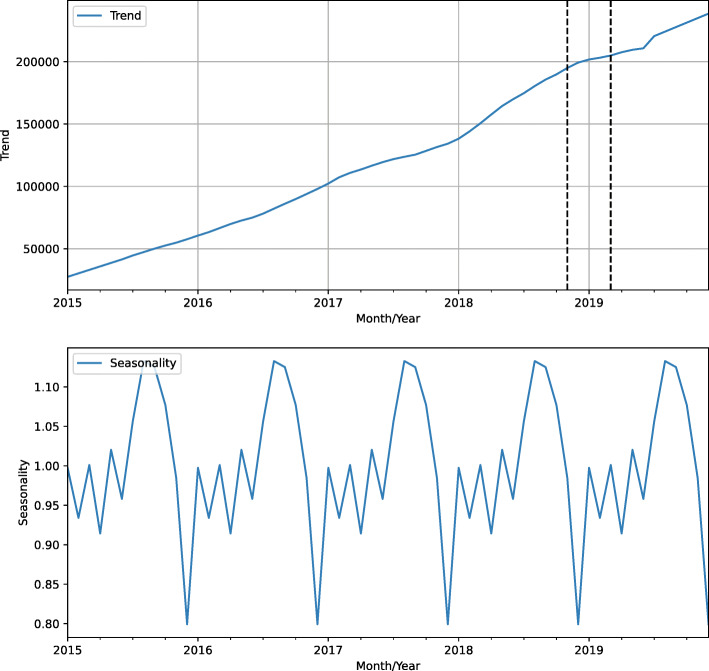
Amount of screening tests decomposed into trend and seasonality

Figure 10 depicts the decomposed data into a trend and seasonality screening test chart. The growing trend has been accentuated since the beginning of 2018. In addition, seasonality shows the drop in the number of tests in December and a peak in October.

Benzathine Penicillin G (BPG) remains the treatment of choice for all stages of syphilis [24] and it is the only antibiotic known to cross the placenta and prevent mother-to-child transmission of syphilis [25].

Table 4 shows the acquired syphilis detection rate (per 100,000 inhabitants), syphilis detection rate in pregnant women, and the incidence rate of congenital syphilis (per 1000 live births) between 2016 and 2019, in addition to the distribution of BPG dose in the same period. In this case, we considered 2016 as baseline, since the penicillin distribution data was available only from 2016 and grouped by year.

**Table 4:** Incidence rates of syphilis in pregnant women, congenital syphilis, and distribution of penicillin in Brazil, between 2016 and 2019. Data source: *SINAN/MoH, **SISMAT/MoH

Year	Born Alive	Incidence rates of syphilis in pregnant women*	Incidence rate of congenital syphilis*	Ratio (relation between syphilis in pregnant women and congenital syphilis)	Acquired syphilis detection rate per 100,000 inhabitants	Benzathine Penicillin Distribution**
2016	2,979,259	12·8	7·1	55·54%	44·5	1,557,000
2017	2,857,800	17·4	8·7	50·07%	59·0	648,800
2018	2,923,535	21·4	9·0	41·88%	75·4	1,279,800
2019	2,944,932	20·8	8·2	39·48%	72·8	1,965,550

Thus, we can observe that in 2016, Brazil received penicillin supply levels higher than in 2018 and nearby 2019. In 2017, there were shortages of benzathine penicillin in Brazil and the world [25–27] and this can have influenced the lower distribution in this year.

Although we observed an increase both in the rates of congenital syphilis in Brazil and syphilis in pregnant women between 2016 and 2018, there was a rise in live births without syphilis in the same period. We highlight the years 2018 and 2019 where the ratio between syphilis in pregnant women and congenital syphilis declined to 41·9% in 2018 and to 39·4% in 2019, even considering the increase in testing rates. This means that in 2019, the probability of a child being born with syphilis was 39·48%, considering his mother was diagnosed with syphilis.

Likewise, acquired syphilis detection rate per 100,000 inhabitants maintained a rise between 2016 (*n* = 44·5), 2017 (*n* = 59·0) and 2018 (75·4), but dropped 2·6 cases per 100,000 inhabitants in 2019 (*n* = 72·8). Despite the small difference in the number of cases (5136 fewer cases in absolute numbers), it is important to note the beginning of the decline in cases, when compared to previous years in which rates continued to rise.

**Fig. 11:**
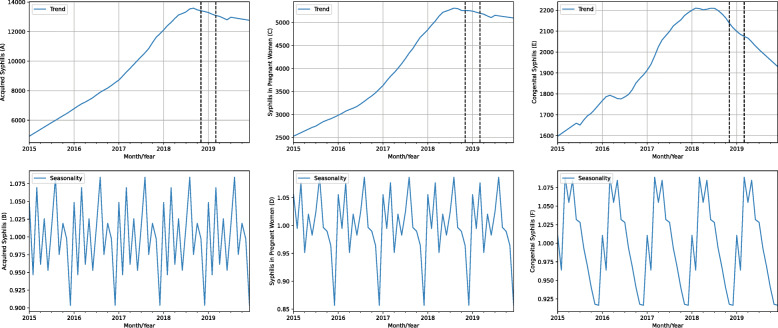
Number of notified cases of acquired syphilis, syphilis in pregnant women and congenital syphilis per month/year decomposed into trend and seasonality

Figure 11 shows the sloped curve relating to the amount of notified cases of acquired syphilis, syphilis in pregnant women and congenital syphilis per month and year, decomposed into trend and seasonality. Analyzing the trends plot, we can observe a discrete decline in the amount of notified cases of acquired syphilis (a) and syphilis in pregnant women (c), with a sharp decline in congenital syphilis (e) cases from the middle of 2018. Regarding the seasonality, we can observe peaks of notified cases in acquired syphilis and syphilis in pregnant women in August (second peak in March), and a drop in December. Congenital syphilis notified cases show peaks in March and May, and drop in December.

These data reveal positive changes in the detection, treatment, and cure of acquired syphilis, syphilis in pregnant women, and congenital syphilis.

## Discussion

Public health campaigns and its impact have been evaluated through several methods in the literature. For instance, Nadarzynski et al. [6] used a qualitative approach to explore young women’s views on sexual health promotion. The group analyzed questionnaires about attitudes towards sexual health, SNS engagement and STI testing orders. This analysis consisted of using standard descriptive statistics including means, standard deviations, and frequencies. Dowshen et al. [8] assessed a youth-driven, social media-based campaign aimed at improving knowledge about and increasing testing for STIs. The researchers (i) tracked website and social media use throughout the campaign, using Google Analytics dashboard, (ii) performed an online survey of attitudes towards STI testing 9 months after campaign launch, and (iii) compared rates of STI testing during the 1-year period immediately prior versus 1-year immediately after campaign launch. These data were analyzed using standard statistics as well. Another study performed by Wakefield et al. [10] sought to assess the impact of several tobacco control policies and televised antismoking advertising on adult smoking prevalence. The authors assessed the effect on smoking prevalence of televised antismoking commercials, cigarette costliness, monthly sales of pharmaceutical products, population exposure to smoke-free laws, and a population survey data, using time series autoregressive integrated moving average (ARIMA) analysis.

These were studies that most used different approaches to evaluate in different aspects how a health campaign could be assessed. However, we should consider that to analyze the reach of a public health campaign, it is necessary to observe its data in a structured and integrated way through different dimensions.

In this study, we presented an inédite campaign data that can help other researchers better analyze and compare public health campaigns. In addition, our framework observed that, starting from June 2018, there was an increasing slope corresponding to the ‘interest over time’ in relation to search activity on the Internet, along with an increase of spontaneous e-news related to syphilis. Twelve MOOCs related to syphilis disease were provided by AVASUS to promote lifelong learning to health professionals, teachers, and students, as well as to the general public, reaching over 22,000 students enrolled until September 2020. Serological tests can demonstrate public awareness of the importance of testing and treatment focusing on the cure, which was the goal of the campaign. In 2019, there was an increase of 375·18% in the number of syphilis serological tests when compared to 2015, even accounting for population growth. Finally, syphilis notification cases show a decreasing curve starting from the middle of 2018. The cases of acquired syphilis and syphilis in pregnant women still showed a slight decrease when comparing 2018 and 2019. Nevertheless, it is important to note that this is the first time that there has been a reduction in reported cases of the disease in recent years, even considering the significant increase in testing in the same period. In addition, cases of congenital syphilis showed a drop of 31·79% in the last year (2019).

Observing these results, it is important to highlight that in the years 2015 to 2017, Brazil had programs to strengthen primary health care. However, syphilis was a neglected sexually transmitted infection in the country. Thus, there were no communication actions in this period to confront the disease, especially where the population and health professionals were concerned.

According to our analysis based on our framework, it is possible to see a positive influence of the “Syphilis No!” Project and its campaigns as inducers of the policies to fight syphilis in Brazil, as it managed to implement a testing, treatment, and cure Agenda. This association is made because in the years before 2018, the campaign did not yet exist. This agenda was guided by award-winning advertising campaigns [28–30] in the country, and also by a health training agenda focusing on syphilis and other sexually transmitted infections.

It is noteworthy that the framework, despite not making an inference based on a causal relationship, allowed the development of structured and integrated analysis of heterogeneous data in a single view. This is an innovative aspect from the perspective of public health policy management, especially when it comes to health promotion agendas, as in the case of the “Syphilis No!” Project.

Another limitation of the study is related to the high number of penicillin doses distributed in 2019 (*n* = 1,965,550). It is difficult to measure the reach with this data, since there was a shortage of penicillin in previous years in Brazil and worldwide. A more precise data for the analysis of syphilis treatment is the use of medication in the primary health care units instead of the distribution of the medication, but this data is unavailable.

In addition, it is interesting to discuss whether those indicators are influencing each other. For example, could MOOCs for health care workers directly influence testing indicators? It is important to pay attention to the Educational dimension and its possible chain reaction that allows to improve the technical work environment when health professionals, professors and students are reached out. Education is a fundamental human right and a transformative process of many other aspects of an individual’s life. MOOCs for health care workers, research and publications can show the interest related to qualification in the academic area.

## Conclusions

The multidimensional analysis framework presented here, and implemented in the Hermes system, is important in this context as it manages to capture the available data, transform them into time series and reflect them in a dashboard that make it possible to analyze public health actions, going beyond the data of epidemiological surveillance. It is worthy of note that, epidemiological data observed in isolation might not sufficiently explain the phenomena pointed out in this analysis.

This framework was applied to “Syphilis No!” campaign and multiple data sources were analyzed over time. Results of this study are primarily exploratory and are important to understand the reach of this public health campaign. According to this analysis, it is possible to see positive changes over time, especially from 2018, in the three dimensions analyzed, enabling policy makers to assess its awareness strategies developed to alert people about health care and attendant behavioral changes.

## Data Availability

The datasets used and/or analyzed during the current study are available from the corresponding author on reasonable request.
